# Regulatory T Cells in Type 1 Autoimmune Pancreatitis

**DOI:** 10.1155/2012/795026

**Published:** 2012-03-28

**Authors:** Kazushige Uchida, Takeo Kusuda, Masanori Koyabu, Hideaki Miyoshi, Norimasa Fukata, Kimi Sumimoto, Yuri Fukui, Yutaku Sakaguchi, Tsukasa Ikeura, Masaaki Shimatani, Toshiro Fukui, Mitsunobu Matsushita, Makoto Takaoka, Akiyoshi Nishio, Kazuichi Okazaki

**Affiliations:** ^1^Department of Gastroenterology and Hepatology, Kansai Medical University, 10-15 Fumisono Cho, Moriguchi, Osaka 570-8506, Japan; ^2^The Third Department of Internal Medicine, Division of Gastroenterology and Hepatology, Kansai Medical University, 2-3-1 Shinmachi, Hirakata, Osaka 573-1191, Japan

## Abstract

Autoimmune pancreatitis (AIP) is a newly recognized pancreatic disorder. Recently, International Consensus Diagnostic Criteria for AIP (ICDC) was published. In this ICDC, AIP was classified into Type 1 and Type 2. Patients with Type 1 AIP have several immunologic and histologic abnormalities specific to the disease, including increased levels of serum IgG4 and storiform fibrosis with infiltration of lymphocytes and IgG4-positive plasmacytes in the involved organs. Among the involved organs showing extrapancreatic lesions, the bile duct is the most common, exhibiting sclerosing cholangitis (IgG4-SC). However, the role of IgG4 is unclear. Recently, it has been reported that regulatory T cells (Tregs) are involved in both the development of various autoimmune diseases and the shift of B cells toward IgG4, producing plasmacytes. Our study showed that Tregs were increased in the pancreas with Type 1 AIP and IgG4-SC compared with control. In the patients with Type 1 AIP and IgG4-SC, the numbers of infiltrated Tregs were significantly positively correlated with IgG4-positive plasma cells. In Type 1 AIP, inducible costimulatory molecule (ICOS)^+^ and IL-10^+^ Tregs significantly increased compared with control groups. Our data suggest that increased quantities of ICOS^+^ Tregs may influence IgG4 production via IL-10 in Type 1 AIP.

## 1. Introduction

In 1961, Sarles et al. observed the first case of idiopathic chronic pancreatitis with hypergammaglobulinemia, in which an autoimmune mechanism was supposedly involved [1]. In 1991, Kawaguchi et al. reported two cases of an unusual lymphoplasmacytic sclerosing inflammatory disease involving the entire pancreas, common bile duct, gallbladder, and, in one patient, the lip [2]. In addition, two patients presented mass-like enlargement of the pancreatic head. Histopathological characteristics included diffuse lymphoplasmacytic infiltration, marked interstitial fibrosis, acinar atrophy, and obliterative phlebitis of the pancreatic and portal veins, which was termed as lymphoplasmacytic sclerosing pancreatitis (LPSP). In 1995, Yoshida et al. first proposed the concept of “autoimmune pancreatitis (AIP),” in which patients showed a diffusely enlarged pancreas, a narrowing pancreatogram, increased serum IgG, the presence of autoantibodies, fibrotic changes with lymphocytic infiltration, and steroidal efficacy [3]. In 2001, Hamano et al. reported that elevated serum IgG4 levels were highly specific and sensitive for the diagnosis of AIP [4]. In 2003, Kamisawa et al. suggested that AIP is a systemic disease, based on the findings that the pancreas and other involved organs have abundant infiltration of IgG4-positive plasma cells [5]. Thereafter, Japanese investigators reported numerous AIP cases, and AIP has been accepted as a new clinical entity [6–9].

Human regulatory T cells (Tregs) were first isolated from peripheral blood and characterized as CD4^+^CD25^high^ T cells by several groups in 2001 [10–12], based on the finding in 1995 that mouse Tregs constitutively express CD25 [13]. We now know that these cells play a critical role in preventing autoimmune diseases by suppressing self-reactive T cells—which are present in all healthy individuals—through incompletely understood mechanisms that involve cell contact and secretion of inhibitory cytokines [14]. Although the role of Tregs in AIP remains unclear, we will discuss Tregs and the mechanism of IgG4 related AIP.

## 2. Type 1 and Type 2 AIP

Reports from Europe [15] and the United States [16] described unique histological patterns in the resected pancreases of patients with mass-forming chronic nonalcoholic pancreatitis with epithelial destruction by granulocytes, which is now supposed to be distinguishable from Type 1 AIP, IgG4-related AIP or LPSP, and called idiopathic duct centric pancreatitis (IDCP), AIP with granulocyte epithelial lesions (AIP with GEL), or Type 2 AIP [17]. In 2011, the International Consensus Diagnostic Criteria for Autoimmune Pancreatitis (ICDC) was published. In this ICDC, AIP was classified into Type 1 and Type 2 [18]. Most of the Japanese AIP cases are LPSP, whereas those concerning IDCP are very few. Although we recently reported the first case of IDCP in Japan with full radiological and histopathological findings [19], it still remains unclear whether the clinical manifestations of the Japanese patients with IDCP are similar to those of Western countries or not. Therefore, Japanese consensus clinical guidelines have focused on Type 1 AIP (IgG4-related AIP) [20–23]. An overlap in the histological features of the two patterns may exist in some patients. Although the pathogenesis is still unclear, the most important issue in managing AIP is to differentiate it from pancreas and biliary malignancy.

## 3. Other Organ Involvement (OOI) in Type 1 AIP

Type 1 AIP often shows other organ involvement (OOI) such as AIP, sclerosing cholangitis, retroperitoneal fibrosis, enlarged celiac and hilar lymph nodes, chronic thyroiditis, and interstitial nephritis [24–28]. Moreover, sialoadenitis is also major complication with Type 1 AIP. The patients with Mikulicz's disease (MD) usually have bilateral, painless, and symmetrical swelling of the lachrymal, parotid, and submandibular glands [29]. This disease is originally classified as an atypical type of Sjögren's syndrome. Recently, MD has been considered to be completely different from Sjögren's syndrome because of the lack of anti-SS-A/Ro or anti-SS-B/La antibodies, elevated serum levels of IgG4, infiltration of IgG4-positive plasma cells into the glands, and recovery of secretion with steroid treatment [25]. Sclerosing cholangitis with Type 1 AIP shows various cholangiographic features similar to those of primary sclerosing cholangitis (PSC). However, the steroid responses and the prognoses of Type 1 AIP patients with sclerosing cholangitis differ from these features in patients with PSC, which suggests different pathogenesis. These findings led us to the concept of ‘‘IgG4-related disease” [30] such as IgG4-related systemic sclerosing disease [5, 31], systemic IgG4-related plasmacytic syndrome (SIPS) [32], and IgG4-positive multiorgan lymphoproliferative syndrome (IgG4-MOLPS) [33].

## 4. Regulatory T Cells (Tregs)

Tregs expressing the transcription factor forkhead box P3 (FOXP3) originally identified by Hori et al. have key roles in the immune system, which are indispensable for the maintenance of dominant self-tolerance and immune homeostasis [34]. Dysfunction of FOXP3 is known to cause fatal autoimmune diseases, immunopathology, and allergy [35]. Most of FOXP3^+^ Tregs are CD4^+^ T cells strongly expressing CD25 (the interleukin-2 (IL-2) receptor *α*-chain), can suppress the activation, proliferation and effector of immune cells, including CD4^+^ and CD8^+^ T cells, natural killer (NK) and NKT cells, B cells, and antigen-presenting cells (APCs) in vitro and in vivo [36]. This unique ability can make FOXP3^+^ Tregs to control immune responses to prevent development of autoimmune disease, immunopathology, and allergy, as well as to maintain allograft tolerance and fetal-maternal tolerance during pregnancy [37].

Increased CD4^+^CD25^+^ T cells were observed in the periphery and inflamed tissues of the patients with rheumatoid arthritis, Sjögren's syndrome, and psoriasis, compared with healthy controls [38]. The increased numbers of Tregs suggest that the reason for failed regulation in the inflamed tissue may be insufficient or defective Tregs function due to either cell-intrinsic or cell-extrinsic factors. However, the precise mechanism is still unclear.

## 5. Subtypes of Regulatory T Cells

CD4^+^ T-cell differentiation into the conventional T-cell and Tregs lineages can be identified by phenotypic markers. All T-cell lineages originate in the thymus and emigrate as naïve CD45RA^+^ T cells, and activation of naive T cells in the periphery induces their differentiation into both conventional and regulatory subsets. Conventional T cells further differentiate into memory T cells, which can be reactivated. CD45RA^+^ naïve Tregs also differentiate into CD45RA^−^ effector Tregs. On the other hand, the CD45RA^−^ peripheral Tregs compartment is converted Tregs-like cells, which are derived from conventional T cells. These converted Tregs-like cells have cell surface marker expression similar to that expressed by natural Tregs [39].

The effector Tregs subsets are heterogeneous in the expression of inducible costimulatory molecule (ICOS) [40]. In the periphery, two functionally different subsets of effector Tregs, ICOS^+^ or ICOS^−^ effector Tregs actively produce the suppressive cytokines IL-10 or TGF-*β*, respectively [40]. It is reported that the growth of Tregs secreting IL-10 required dendritic cells (DCs) expressing high-level ICOS-ligand and was prevent by blockade of ICOS-ICOS-ligand signaling [41]. DC is important in the proliferation of ICOS^+^ or ICOS^−^ Tregs. While activated plasmacytoid DCs (pDCs) preferentially promote the proliferation of the ICOS^+^ Tregs through ICOS-ligand, activated myeloid DCs (mDCs) preferentially promote the proliferation of the autologous ICOS^−^ Tregs through B7 signaling [40].

## 6. Tregs in Type 1 AIP

### 6.1. Flow Cytometric Analysis of Peripheral Blood in the Patient with Type 1 AIP

To clarify the role of Tregs in IgG4-related diseases, we analyzed Tregs showing CD4^+^CD25^high^ and CD4^+^CD25^+^CD45RA^+^ (naïve) from peripheral blood by flow cytometry in the patients with Type 1 AIP. For comparison, we also analyzed patients with other pancreatic disease (idiopathic or alcoholic pancreatitis) and healthy subjects as controls. In Type 1 AIP, in addition to increased soluble CTLA4, circulatory naïve (CD45RA^+^) Tregs were significantly decreased in the peripheral blood of the patients with AIP, whereas the major population of effector (CD45RA^−^) Tregs was significantly increased [42]. In addition, we studied infiltrating cells in the pancreas by immunohistochemistry and analyzed ICOS^+^ Tregs and IL-10^+^ Tregs in the peripheral blood by flow cytometry. ICOS^+^ Tregs were significantly higher in AIP patients than in the patients with other pancreatic diseases and the healthy control group. IL-10^+^ Tregs were significantly higher in AIP patients than in the healthy control group [43]. We previously investigated three types of DCs: mDC (Lin-HLA-DR^+^CD11c^+^), pDC (Lin-HLA-DR^+^CD11c^−^), and CD123^+^ pDC (Lin-HLA-DR^+^CD11c^−^CD123^+^). There were no significant differences in the three kinds of DCs among the healthy control, alcoholic and idiopathic CP, and AIP groups. However, mDC and CD123^+^ pDC significantly decreased in patients with AIP with steroid therapy, compared with other groups [41]. The relationship between DC and Tregs in AIP is still unclear.

### 6.2. Immunohistochemical Analysis in Type 1 AIP

IgG4-related sclerosing cholangitis (SC) was recognized as a disease entity characterized by sclerosing inflammation with abundant IgG4-positive plasma cells; Type 1 AIP was associated in most cases. We analyzed Tregs in the pancreas and liver of Type 1 AIP. In the pancreas of Type 1 AIP, the ratio of Foxp3-positive cells to infiltrated mononuclear cells (Foxp3/Mono) was significantly higher than in patients with alcoholic chronic pancreatitis. In Type 1 AIP, Foxp3/Mono and IgG4/Mono were positively correlated. This data is similar to flow cytometric analysis.

We compared the laboratory and immunohistochemical findings of the liver biopsy specimens in the patients with IgG4-SC and primary sclerosing cholangitis (PSC). After staining these specimens with anti-IgG1, anti-IgG4, and anti-Foxp3 antibodies, we compared the ratio of IgG4-, IgG1-, and Foxp3-positive cells to infiltrated mononuclear cells (IgG4, IgG1, Foxp3/Mono) among specimens. The ratio of IgG4/G1-positive plasma cells was significantly higher in IgG4-SC than in PSC. The Foxp3/Mono ratio in patients with IgG4-SC was significantly increased compared with PSC. In the patients with IgG4-SC, Foxp3-positive cells were significantly correlated with IgG4-positive cells. In the other groups, there was no correlation [44].

## 7. Animal Models of Type 1 AIP

We previously reported several autoantibodies in the patients with Type 1 AIP. Among the 26 patients with Type 1 AIP, antilactoferrin (LF) was detected in the sera of 19 (73.1%), antinuclear antibody (ANA) in 18 (69.2%), anticarbonic anhydrase (CA)-II in 14 (53.8%), RF in 6 (23.1%), antismooth muscle antibody in 4 (15.4%), antiglutamic acid decarboxylase antibody in 1 (3.8%), and antiislet cell antibody in 1 (3.8%). However, AMA was not found in the sera of any of these patients [45]. We hypothesized that LF or CA-II may be one of the candidates of target antigen in Type 1 AIP. We established animal models using LF or CA-II as antigen. In neonatal thymectomized (nTx)-BALB/c mouse models immunized with CA-II or LF, CD4^+^ T cells, rather than B cells, there are the predominant infiltrates in pancreas and salivary gland, and around bile duct, which are similar to human Type 1 AIP [46]. The results from these animal models suggest that the depletion of thymus-derived Tregs in the periphery [46] and major histocompatibility complex (MHC) class II restricted autoreactive CD4^+^ T cells that escape from positive selection in the thymus may be of importance to the induction of target organs. In the early stage of Type 1 AIP, these CD4^+^ T cells probably induce proinflammatory reactions as direct cytotoxic effects through Fas ligand [47]. In the MHC class II-deficient mouse [48] and in WBN/Kob rat models [49], CD8^+^ T cells may play roles as effector cells. WBN/Kob rats spontaneously develop sialoadenitis, thyroiditis, sclerotic cholangitis, and tubulointestinal nephritis because of congenitally decreased peripheral Tregs. CD8^+^ T cells also seem to be effector cells in spite of unknown target antigens. WBN/Kob rats showed deposition of tissue-specific IgG2b in the injured pancreas and lachrymal glands [49]. Although details of the IgG subclass in rodents remain unclear, rat IgG2b, a minor subclass of IgG, is separated in a similar position to human IgG4 by electrophoresis. In view of the results from animal models, even though CD8^+^ T cells may be partially involved, CD4^+^ T cells play major roles in the development of systemic inflammation, which are similar to the lesions in human IgG4-related diseases [9, 50]. As TGF-*β* is an important regulatory factor in maintaining immune homeostasis, TGF-*β*-dominant negative mutant mice suggest that the loss of TGF-*β* signaling may contribute to AIP [51].

## 8. Our Hypothesis for the Pathogenesis of AIP as IgG4-Related Disease

**Figure 1:**
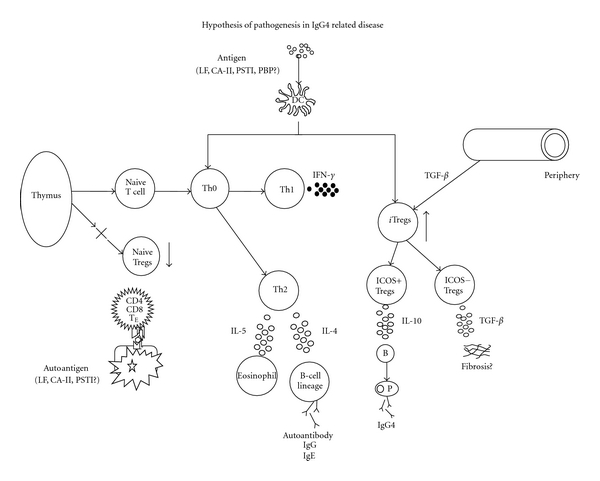
Hypothesis for the pathogenesis of Type 1 autoimmune pancreatitis (AIP) in IgG4-related disease. In regard to central tolerance, naïve and natural regulatory T cells (Tregs) derived from the thymus suppress autoreactive CD4 or CD8 cells in the normal state. In IgG4-related disease, the basic concept is a biphasic mechanism of “induction” and “progression”. Initial response to self-antigens (e.g., lactoferrin (LF), carbonic anhydrase II (CA-II), CA-IV, pancreatic secretory trypsin inhibitor (PSTI), amylase-alpha, and plasminogen binding protein (PBP) peptide of *Helicobacter pylori*) might be induced by decreased naïve Tregs. Th2 immune responses are followed by a Th1-type immune response with the release of proinflammatory cytokines (interferon-*γ* (IFN-*γ*), interleukin (IL)-1*β*, IL-2, tumor necrosis factor-*α* (TNF-*α*)). Th2-type immune responses, producing IgG, IgG4, and autoantibodies may be involved in the pathophysiology of progression. The production of IgG4 may be regulated by increased IL-10 secreted from inducible co-stimulatory molecule (ICOS)^+^ effector Tregs. Fibrosis may be regulated by TGF-*β* secreted from ICOS^−^ Tregs. This figure modified from Okazaki et al. [54]. DC: dendritic cell, TE: effector T cell, nTreg: natural Tregs.

Zen et al. reported that Th2 and regulatory cytokines, and Tregs had an important role in IgG4-SC [52]. In the patients with asthma, high-dose allergen exposure during immunotherapy results in both immune deviation of Th2 responses in favor of a Th0/Th1 response and in the generation of IL-10- and TGF-*β*-producing Tregs. Additionally, these cytokines induce preferential switching of B-cell responses in favor of IgG and IgG4 antibodies [53]. We proposed a hypothesis for the pathogenesis of Type 1 AIP (Figure 1). This concept is based on a biphasic mechanism of “induction” and ‘‘progression.” An initial response to self-antigens (e.g., LF, CA-II, CA-IV, pancreatic secretory trypsin inhibitor (PSTI), amylase-alpha, and plasminogen binding protein (PBP) peptide of *Helicobacter pylori*) might be induced by decreased naïve Tregs.

Decreased naive Tregs may be pathogenetic. In the early stage of Type 1 AIP, proinflammatory cytokines (IFN-*γ*, IL-1-*β*, IL-2, TNF-*α*) are released via Th1-type immune response. In the chronic stage, IgG, IgG4, and autoantibodies are produced via Th2-type immune responses. Particularly, increased numbers of ICOS^+^ Tregs may affect IgG4 production via IL-10 in Type 1 AIP. On the other hand, fibrosis may be regulated by TGF-*β* secreted from ICOS^−^ Tregs.

## 9. Conclusion

In conclusion, Tregs seem to be important in the production of IgG4 as well as the induction of IgG4-related disease. However, further studies are necessary to clarify the pathogenesis, including genetic backgrounds, disease-specific antigens, and the role of IgG4.
